# Ovarian carcinosarcoma (malignant mixed Müllerian tumor, MMMT) in a premenopausal woman: a diagnostic challenge—a case report

**DOI:** 10.1186/s13256-026-06253-y

**Published:** 2026-06-26

**Authors:** Gaowa Ailun, Yuefei Li, Dawei Zhang, Yangxing Fu, XiFeng Su, Congxiang Yu

**Affiliations:** 1 Affiliated Hospital of Inner Mongolia Medical University, Hohhot, Inner Mongolia China; 2 Women and Children’s Health Hospital of Inner Mongolia Autonomous Region, Hohhot, Inner Mongolia China; 3 University of Alberta, Edmonton, AB Canada; 4 Inner Mongolia Channel Street Community Health Center, Hohhot, Inner Mongolia China

**Keywords:** Malignant mixed mesodermal tumor, Ovarian neoplasm, Young patient, Diagnostic challenges, Case report

## Abstract

**Background:**

Ovarian carcinosarcoma, also known as malignant mixed Müllerian tumor (MMMT), is a rare and highly aggressive gynecologic malignancy characterized by the presence of both epithelial and mesenchymal components. It predominantly affects postmenopausal women, and its occurrence in premenopausal patients is uncommon. Due to its rarity, optimal management strategies remain poorly defined.

**Case presentation:**

We report the case of a 33-year-old premenopausal Han Chinese woman diagnosed with ovarian carcinosarcoma who underwent complete cytoreductive surgery followed by platinum-based chemotherapy. Germline BRCA1/2 mutation testing and homologous recombination deficiency (HRD) assessment were performed and yielded negative results. Following a favorable response to chemotherapy, maintenance therapy with Olaparib was initiated as an individualized, off-label approach after multidisciplinary team discussion and with full patient-informed consent. Sustained disease control was observed during a 46-month follow-up period, without significant treatment-related adverse events.

**Conclusion:**

This case highlights the importance of accurate diagnosis and optimal surgical and systemic management in ovarian carcinosarcoma. While maintenance therapy with Olaparib was used in an individualized, off-label setting despite negative BRCA and HRD testing, this approach should not be generalized. Further studies are needed to better define the role of targeted therapies in this rare and aggressive tumor subtype.

## Introduction

Ovarian carcinosarcoma (malignant mixed Müllerian tumor, MMMT), also referred to as carcinosarcoma, is a highly aggressive malignant neoplasm characterized by the presence of both epithelial (carcinomatous) and mesenchymal (sarcomatous) components within the same tumor. According to the World Health Organization (WHO) classification, MMMT is considered a metaplastic carcinoma rather than a true sarcoma, reflecting its epithelial origin with secondary mesenchymal differentiation [1, 2].

MMMTs most commonly arise in the uterus, accounting for a small but clinically significant proportion of uterine malignancies. Ovarian involvement is rare, representing less than 5% of all MMMTs, and is associated with an aggressive clinical course and poor prognosis [3, 4]. These tumors predominantly occur in postmenopausal women, with peak incidence reported in the sixth and seventh decades of life [5].

Clinically, ovarian carcinosarcomas (MMMTs) often present with nonspecific symptoms, such as abdominal distension or pelvic pain, and may also be detected incidentally during imaging examinations. Radiological findings typically demonstrate large cystic–solid adnexal masses with heterogeneous components, which are often indistinguishable from other primary ovarian malignancies, particularly epithelial ovarian carcinoma. Serum tumor markers, including CA125, may be elevated but lack specificity, further complicating the preoperative diagnosis [4].

Due to their rarity, nonspecific clinical manifestations, and overlapping imaging features, ovarian carcinosarcomas (MMMTs) pose significant diagnostic challenges, especially in premenopausal women, in whom malignant ovarian tumors are less commonly suspected. As a result, definitive diagnosis frequently relies on postoperative histopathological and immunohistochemical evaluation rather than preoperative clinical or radiological assessment alone [1].

In this report, we describe a rare case of ovarian carcinosarcoma (MMMT) in a 33-year-old premenopausal woman, emphasizing the diagnostic challenges encountered and discussing the multidisciplinary management approach in the context of current literature.

### Case presentation

#### Patient information

A 33-year-old Han Chinese premenopausal woman with no significant past medical history was incidentally found to have a pelvic mass during a routine health examination. She was nulligravid and reported no gynecological symptoms, including abdominal pain, abnormal vaginal bleeding, gastrointestinal discomfort, or urinary complaints. There was no personal or family history of gynecologic malignancy or hereditary cancer syndromes.

#### Clinical findings

On physical and gynecological examination, the external genitalia and vagina appeared normal. The cervix was of normal size and appearance, with no visible lesions or abnormal discharge. Bimanual pelvic examination revealed a large cystic–solid mass occupying the pelvic cavity, estimated to be approximately 20 cm in diameter. The mass was firm, poorly mobile, and non-tender. Due to its size, detailed assessment of the uterus and adnexa was limited.

### Diagnostic imaging and laboratory investigations

#### Diagnostic imaging

**Fig. 1:**
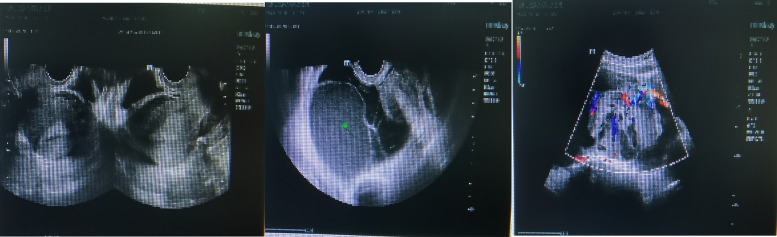
Uterus: anteverted, pear-shaped, measuring 52 × 47 × 46mm; endometrial thickness 12 mm; homogenous myometrial echotexture pelvic mass: cysticsolid, measuring 190 × 124×210mm; internal echoes with fine punctate echoes and septations; irregular solid components; ovaries not visualized impression: cysticsolid pelvic mass, suggestive of cystadenoma

Initial pelvic ultrasonography revealed an anteverted, pear-shaped uterus with homogeneous myometrial echotexture and a normal endometrial thickness. A large cystic–solid pelvic mass was identified, characterized by internal echoes, septations, and irregular solid components. Both ovaries were not visualized due to compression and obscuration by the mass. Based on these sonographic findings, the lesion was considered suspicious for an adnexal neoplasm, and malignancy could not be excluded (Fig. 1).

Pelvic magnetic resonance imaging (MRI) was subsequently performed for further characterization. MRI demonstrated a large cystic–solid mass occupying the lower abdomen and pelvic cavity, with heterogeneous signal intensity, multiple septations, and soft tissue nodules. Progressive enhancement of the solid components and focal omental thickening were observed. The uterus and cervix demonstrated normal signal intensity without evidence of intrauterine mass or endometrial thickening. These imaging features favored a diagnosis of primary ovarian malignancy, particularly ovarian mucinous cystadenocarcinoma, while metastatic disease could not be definitively excluded.

**Fig. 2:**
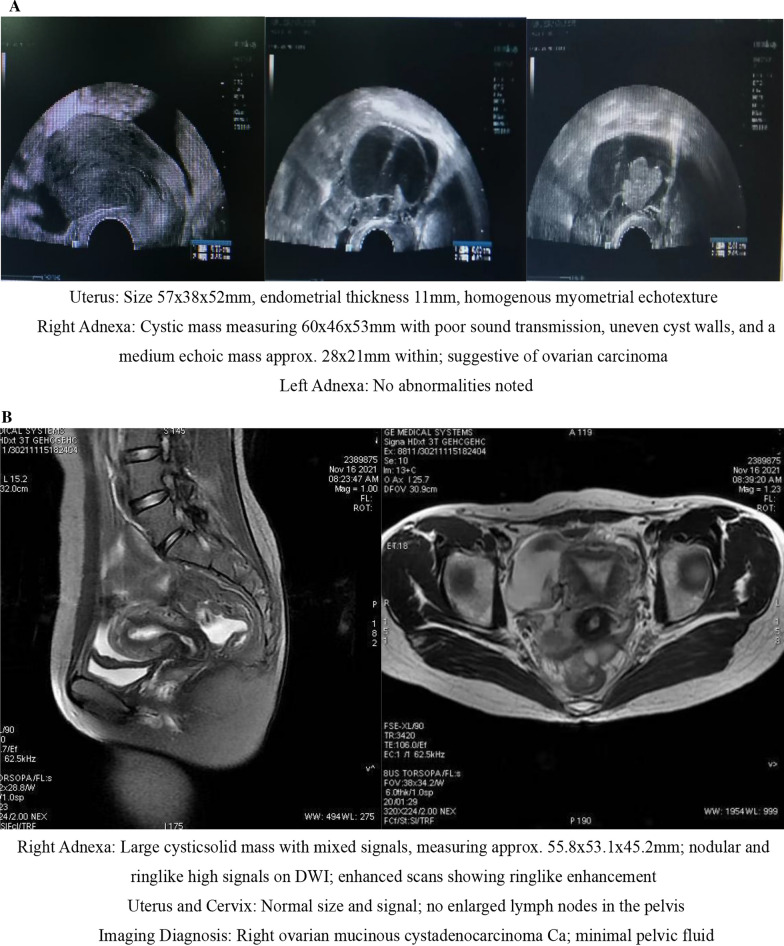
MRI Pelvis. Large cysticsolid mass with mixed signal intensity (209 × 189 × 136mm) in the lower abdomen and pelvis; multiple septations and soft tissue nodules; well-defined borders; progressive enhancement in solid components; omental thickening anterior to the mass, significantly enhancing uterus and cervix: normal signal intensity; no enlarged lymph nodes in the pelvis imaging diagnosis: ovarian mucinous cystadenocarcinoma Ca; focal omental thickening, possible metastasis not excluded

Overall, although imaging studies strongly suggested a malignant ovarian tumor, the radiological findings were nonspecific and did not allow definitive differentiation between epithelial ovarian carcinoma and rare ovarian malignancies, highlighting the limitations of preoperative imaging in establishing a precise histological diagnosis (Fig. 2).

#### Laboratory investigations

Preoperative laboratory evaluation revealed elevated serum tumor markers, including cancer antigen 125 (CA125, 102.60 U/mL), cancer antigen 19-9 (CA19-9, 101.53 U/mL), and human epididymis protein 4 (HE4, 95.70 pmol/L), supporting the suspicion of an ovarian malignancy. No ascites was detected on imaging, and routine laboratory tests, including stool examination and gastroscopy, were unremarkable.

Although additional tumor markers such as carcinoembryonic antigen (CEA) and serum *β*-human chorionic gonadotropin (*β*-HCG) may be included in the evaluation of adnexal masses in younger women, their measurement is typically guided by clinical presentation and imaging characteristics. In this case, there were no gastrointestinal symptoms or imaging features suggestive of metastatic gastrointestinal malignancy to warrant routine CEA assessment. Similarly, serum *β*-HCG is primarily useful in the evaluation of germ cell tumors or pregnancy-related conditions. The absence of radiological features consistent with ovarian germ cell tumors and the lack of clinical suspicion of pregnancy made *β*-HCG measurement unnecessary in the initial diagnostic workup. Therefore, the diagnostic strategy focused on ovarian cancer-associated markers in conjunction with imaging findings to guide surgical decision-making.

#### Diagnostic considerations

Taken together, the imaging findings and laboratory results indicated a high likelihood of malignant ovarian disease; however, they lacked specificity for malignant mixed mesodermal tumor. Consequently, a definitive diagnosis could only be established through postoperative histopathological and immunohistochemical examination.

#### Clinical impression and differential diagnosis

Based on the combination of physical findings, imaging characteristics, and tumor marker elevation, the leading preoperative diagnosis was primary ovarian malignancy, with ovarian mucinous cystadenocarcinoma considered most likely. Other differential diagnoses included borderline ovarian tumors and rare ovarian sarcomatous neoplasms. Given the diagnostic uncertainty and the size of the mass, surgical exploration was deemed necessary for definitive diagnosis and treatment.

### Therapeutic intervention

#### Surgical management

The patient initially underwent laparoscopic exploration with left adnexectomy on September 30, 2021, for removal of the pelvic mass. Histopathological examination revealed features consistent with malignant mixed mesodermal tumor. Following multidisciplinary discussion, definitive surgical staging was performed on November 23, 2021, including total hysterectomy, right adnexectomy, omentectomy, and pelvic and para-aortic lymphadenectomy, aiming for maximal cytoreduction.

#### Pathological findings

**Fig. 3:**
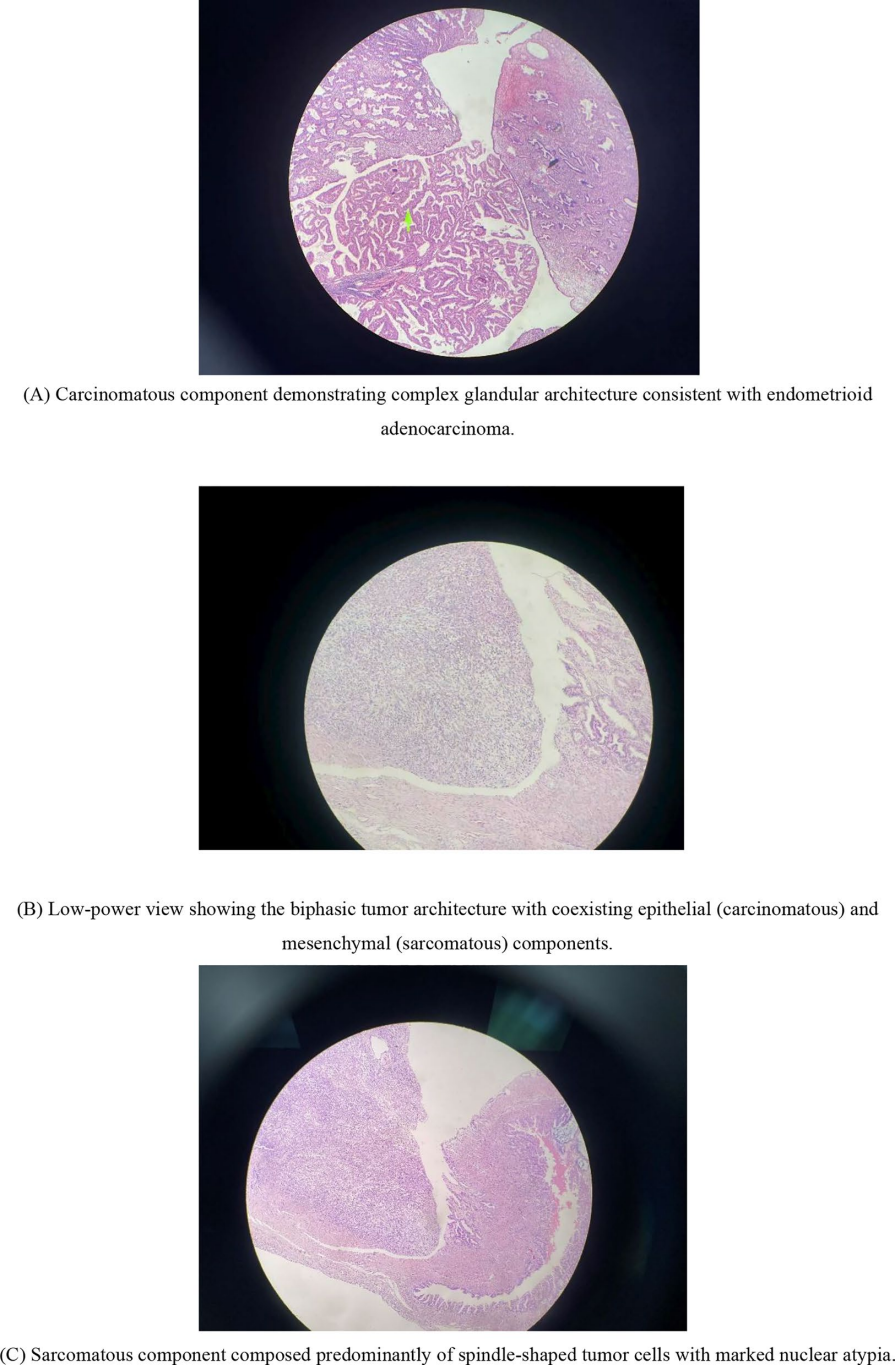
Histopathological features of primary ovarian carcinosarcoma (hematoxylin and eosin staining). **A** Carcinomatous component demonstrating complex glandular architecture consistent with endometrioid adenocarcinoma. **B** Low-power view showing the biphasic tumor architecture with coexisting epithelial (carcinomatous) and mesenchymal (sarcomatous) components. **C** Sarcomatous component composed predominantly of spindle-shaped tumor cells with marked nuclear atypia

Histopathological evaluation confirmed the diagnosis of ovarian malignant mixed mesodermal tumor, composed of high-grade endometrioid adenocarcinoma with sarcomatoid components (Fig. 3). The tumor involved both ovaries on final pathological assessment. Gross and microscopic evaluation of the hysterectomy specimen showed no evidence of primary endometrial carcinoma or carcinosarcoma, and the endometrial cavity was free of tumor involvement, supporting a primary ovarian origin. Immunohistochemical analysis demonstrated AE1/AE3 (focally positive), ER (negative), CK7 (focally positive), CK20 (negative), P53 (negative), Ki-67 labeling index was approximately 50%, P16 (negative), PR (negative), Vimentin (positive). These findings support the biphasic nature of the tumor, consistent with ovarian carcinosarcoma (MMMT). Based on comprehensive surgical staging and final histopathological findings, the tumor was classified as FIGO stage IB primary ovarian carcinosarcoma (2014 FIGO), with disease confined to both ovaries and no evidence of extra-ovarian pelvic extension, lymph node metastasis, or distant spread.

#### Adjuvant therapy and follow-up

Postoperatively, the patient received six cycles of adjuvant chemotherapy with paclitaxel and carboplatin. A venous access port was implanted to facilitate chemotherapy administration. Following completion of chemotherapy, maintenance therapy with Olaparib was initiated. The patient tolerated treatment well, with no severe adverse events reported.

**Fig. 4:**
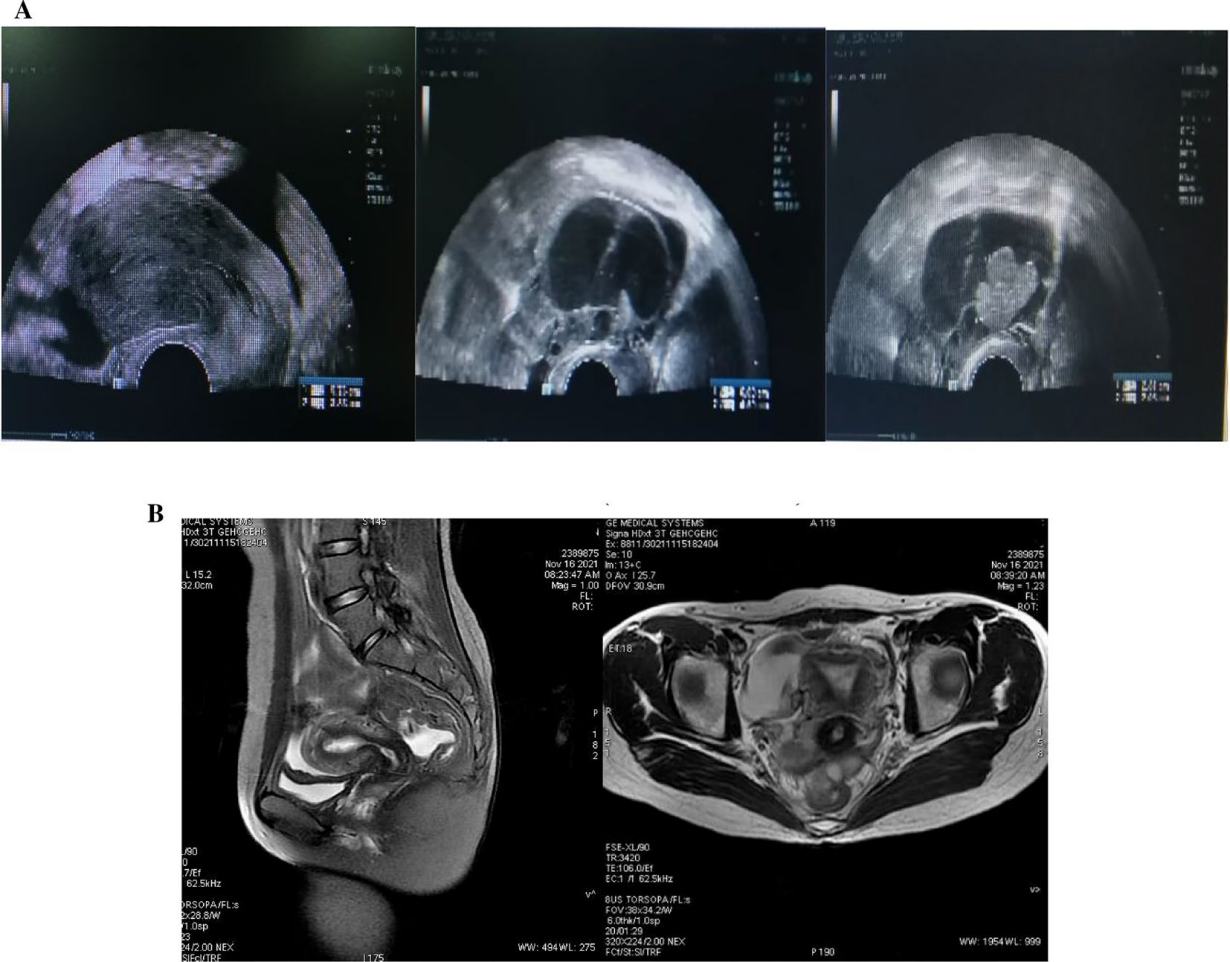
MRI Pelvis (Second Visit). **A** Uterus: size 57 × 38 × 52mm, endometrial thickness 11 mm, homogenous myometrial echotexture. Right adnexa: cystic mass measuring 60 × 46 × 53mm with poor sound transmission, uneven cyst walls, and a medium echoic mass approx. 28 × 21 mm within; suggestive of ovarian carcinoma. Left adnexa: no abnormalities noted. **B** Right adnexa: large cysticsolid mass with mixed signals, measuring approx. 55.8 × 53.1 × 45.2 mm; nodular and ringlike high signals on DWI; enhanced scans showing ringlike enhancement. Uterus and cervix: normal size and signal; no enlarged lymph nodes in the pelvis. Imaging diagnosis: right ovarian mucinous cystadenocarcinoma Ca; minimal pelvic fluid

During follow-up, serum tumor markers showed a significant decline, and subsequent imaging studies, including computed tomography (CT) and pelvic ultrasound, demonstrated no evidence of disease recurrence. The patient remains under regular surveillance. At the latest follow-up in January 2026 (approximately 46 months after completion of chemotherapy and 45 months after initiation of Olaparib), there was no clinical, biochemical, or radiological evidence of recurrence (Fig. 4A, B).

#### Treatment challenges and ethical considerations

The primary treatment challenge in this case was the rarity of ovarian carcinosarcoma (MMMT) and the absence of standardized treatment guidelines for premenopausal patients. While treatment strategies were largely guided by protocols for epithelial ovarian carcinoma, individualized considerations were required due to the tumor’s biphasic nature.

Prior to initiating maintenance therapy, the patient was counseled regarding the potential benefits and uncertainties associated with Olaparib treatment, including the possibility of limited efficacy and unknown long-term outcomes in MMMT. Written informed consent was obtained, and the patient agreed to close clinical monitoring throughout therapy.

## Discussion

Ovarian carcinosarcoma, historically referred to as malignant mixed Müllerian tumor (MMMT), is a rare and highly aggressive malignancy characterized by the coexistence of malignant epithelial and mesenchymal components. It accounts for less than 1% of all ovarian malignancies and is associated with poor prognosis due to its advanced stage at diagnosis, aggressive biological behavior, and high recurrence rate. Most reported cases occur in postmenopausal women, making its occurrence in premenopausal patients particularly uncommon and clinically noteworthy.

The histogenesis of ovarian carcinosarcoma remains controversial. Current evidence supports the monoclonal theory, suggesting that both epithelial and sarcomatous components originate from a single epithelial clone undergoing epithelial–mesenchymal transition. This theory is supported by shared molecular alterations between the two components, which may have implications for therapeutic strategies targeting epithelial tumor biology.

Due to the rarity of ovarian carcinosarcoma, standardized treatment guidelines are lacking, especially for premenopausal patients. Surgical cytoreduction remains the cornerstone of management, with optimal debulking consistently associated with improved survival outcomes. Adjuvant platinum-based chemotherapy is commonly extrapolated from treatment paradigms for high-grade epithelial ovarian cancer, although its efficacy in carcinosarcoma is supported primarily by retrospective studies and small case series rather than prospective trials.

The role of targeted therapies in ovarian carcinosarcoma remains incompletely defined. In particular, the application of poly (ADP-ribose) polymerase (PARP) inhibitors has been well-established in BRCA-mutated epithelial ovarian cancer but remains investigational in carcinosarcoma. In the present case, germline BRCA1/2 mutation testing and homologous recombination deficiency (HRD) assessment were performed and yielded negative results. Despite the absence of these molecular predictors, Olaparib was administered as an individualized, off-label maintenance therapy following platinum-based chemotherapy. This decision was based on the patient’s platinum-sensitive disease, favorable response to chemotherapy, complete cytoreductive surgery, and multidisciplinary team discussion. The investigational nature of this approach was discussed in detail with the patient, and informed consent was obtained prior to treatment initiation.

Available evidence regarding the use of PARP inhibitors in ovarian carcinosarcoma is limited and largely confined to isolated case reports and small retrospective observations. A small number of published cases have described disease stabilization or prolonged progression-free survival following PARP inhibitor therapy, even in the absence of confirmed BRCA mutations, suggesting that platinum sensitivity itself may reflect underlying defects in DNA damage repair pathways beyond classical BRCA-mediated mechanisms. However, these observations remain hypothesis-generating and should be interpreted with caution.

In comparison with previously published reports, the present case is notable for the patient’s premenopausal status, achievement of complete cytoreduction, and prolonged disease control following individualized maintenance therapy. Most reported cases of ovarian carcinosarcoma involve postmenopausal patients with advanced-stage disease and suboptimal surgical outcomes. The favorable clinical course observed in this patient may, therefore, reflect a combination of early intervention, optimal surgical management, platinum sensitivity, and close follow-up rather than the effect of any single therapeutic modality.

Importantly, the use of Olaparib in this case should not be interpreted as evidence supporting routine PARP inhibitor therapy in BRCA- or HRD-negative ovarian carcinosarcoma. Instead, this report highlights the potential role of individualized treatment strategies in selected patients when standard therapeutic options are limited. Further prospective studies and collaborative registries are required to better define the molecular landscape of ovarian carcinosarcoma and to clarify the potential role of targeted therapies in this rare and aggressive tumor subtype [6, 7].

## Conclusion

Ovarian carcinosarcoma is a rare and aggressive malignancy with limited evidence to guide optimal management, particularly in premenopausal patients. This case highlights the importance of accurate histopathological diagnosis, optimal cytoreductive surgery, and platinum-based chemotherapy as the foundation of treatment. In selected cases, individualized therapeutic approaches may be considered when standard options are limited.

In the present case, maintenance therapy with Olaparib was administered as an off-label, individualized strategy following a favorable response to platinum-based chemotherapy, despite negative BRCA and HRD testing. This approach was undertaken after multidisciplinary discussion and with full patient-informed consent. While a prolonged period of disease control was observed, this outcome should not be generalized, and PARP inhibitor therapy cannot be recommended as standard treatment for BRCA- or HRD-negative ovarian carcinosarcoma based on current evidence.

This report contributes to the limited literature on ovarian carcinosarcoma and underscores the need for further collaborative studies to better define the molecular characteristics of this rare tumor and to clarify the potential role of targeted therapies within carefully selected patient populations.

## Data Availability

All data generated or analyzed during this study are included in this published article and its supplementary information files.
